# Comparing AGS Beers 2019, STOPP version 2, and EU(7)-PIM list in Portuguese older adults in primary health care

**DOI:** 10.1007/s00228-024-03633-5

**Published:** 2024-02-06

**Authors:** Daniela A. Rodrigues, Maria Teresa Herdeiro, Ramona Mateos-Campos, Adolfo Figueiras, Fátima Roque

**Affiliations:** 1 Research Laboratory on Epidemiology and Population Health, Polytechnic of Guarda (IPG), 6300-559 Guarda, Portugal; 2 Health Sciences Research Centre, University of Beira Interior (CICS-UBI), 6200-506 Covilhã, Portugal; 3 PhD Student, University of Salamanca, 37007 Salamanca, Spain; 4 Department of Medical Sciences, Institute of Biomedicine (iBiMED), University of Aveiro, 3810-193 Aveiro, Portugal; 5 Area of Preventive Medicine and Public Health, Department of Biomedical and Diagnostic Sciences, University of Salamanca, 37007 Salamanca, Spain; 6 Department of Preventive Medicine and Public Health, University of Santiago de Compostela, 15702 Santiago de Compostela, Spain; 7 Health Research Institute of Santiago de Compostela (IDIS), 15706 Santiago de Compostela, Spain; 8 Consortium for Biomedical Research in Epidemiology and Public Health (CIBER Epidemiology and Public Health-CIBERESP), 28001 Madrid, Spain

**Keywords:** Potentially inappropriate medications, Older adults, Primary health care, AGS 2019 Beers criteria, STOPP v2 criteria, EU(7)-PIM list

## Abstract

**Purpose:**

This study aims to identify PIM prevalence in older adults according to the 2019 Beers criteria, Screening Tool of Older Person’s Prescriptions version 2 (STOPP v2) criteria, and the Portuguese EU(7)-PIM list and also to analyze the concordance between these criteria.

**Methods:**

A retrospective study was conducted among 1200 Portuguese older adults (≥ 65 years old), users of primary health care. Demographic, clinical, and pharmacological data were collected concerning the period between April 2021 and August 2022. A comparative analysis was performed between the three PIM identification criteria, and the concordance was determined according to the Lin concordance correlation coefficient.

**Results:**

The mean age was 76.3 (SD 7.7) years old and 57.6% of the older adults were females. Our findings indicate varying prevalence rates among these criteria with 63.8% (95% CI 61.0–66.6%), 66.8% (95% CI 64.1–69.5%), and 50.1% (95% CI 47.2–53.0%) of the older adults take at least one PIM according to the EU(7)-PIM list, Beers 2019, and STOPP v2 criteria, respectively. The highest prevalence observed was for proton pump inhibitors according to EU(7)-PIM list (30.1%, 95% CI 27.6–32.9) and Beers criteria (30.1%, 95% CI 27.6–32.9) and alprazolam according to STOPP v2 criteria (10.1%, 95% CI 8.4–11.9%). A poor concordance between criteria was observed (< 0.834). The highest concordance coefficient was found between the EU(7)-PIM list and the Beers criteria (0.833), and the lowest between the EU(7)-PIM list and STOPP criteria (0.735).

**Conclusion:**

This study reveals varying prevalence rates of PIM in older adults, as assessed by different criteria, and highlights the need for targeted interventions and improved prescribing practices. In the future, studies should focus on the occurrence of negative outcomes in older adults associated with PIM consumption.

**Supplementary Information:**

The online version contains supplementary material available at 10.1007/s00228-024-03633-5.

## Introduction

Medicines are the most common medical intervention, and its consumption is an important and fundamental component of older adults’ care [1]. However, some medications may become potentially inappropriate medications (PIM) in older people [2], when the risk of adverse effects exceeds the clinical benefit [2, 3]. In fact, it was shown that PIM and frailty interact with each other, having a bidirectional association [4].

Over the last years, there is an increase on the development of interventions to support prescribing and medication use in older people [5–7], and several criteria for PIM identification have been developed. The first published criteria were the Beers criteria in 1991 [8], updated in 2002 [9], becoming American Geriatrics Society (AGS) responsibility in 2011, and updated again in 2012 [10], 2015 [11], 2019 [12], and 2023 [13]. On the other hand, the Screening Tool of Older Person’s Prescriptions (STOPP) criteria were created in 2008 according to European prescription standards [14], with the version 2 (v2) being published in 2015 [15] and version 3 (v3) in 2023 [16]. Also in 2015, a panel of experts from seven European countries (Germany, Finland, Estonia, Holland, France, Spain, and Sweden) developed the EU(7)-PIM list, an explicit criteria tool that allows the identification and comparison of PIM in these countries [17]. Beers criteria were operationalized in Portugal in 2008 [18]. In 2020, the EU(7)-PIM list was operationalized for the Portuguese reality [19], and the STOPP criteria in 2022 [20]. The Beers and STOPP criteria were the basis for developing most of the other criteria that currently exist [3].

According to a recent systematic review, the worldwide overall pooled prevalence of PIM use was 36.7%, varying between 47.0% in Africa, 46.9% in South America, 37.2% in Asia, 35.0% in Europe, 29.0% in North America, and 23.6% in Oceania [21]. Moreover, high PIM prevalence could impose a high economic burden on the older population and society [2]. Primary health care is responsible for the first contact between patients and the health care system and where the most prescriptions for older adults occur [22–24], so research on the use of PIM in this setting is of great interest. One systematic review performed in primary care settings identified factors that contribute to potentially inappropriate prescriptions among older adults which include a greater number of medications and a higher number of comorbidities, while physical comorbidities and psychiatric comorbidities were identified as patient-related clinical risk factors [25]. In Portugal, according to the 2015 Beers Criteria, PIM prevalence in primary care was 68.6% [26]. A nationwide study identified a PIM-defined daily dose frequency of 9.2% according to the EU(7)-PIM list, which was relatively higher in the center region [27]. According to the EU(7)-PIM list and the Beers criteria, PIMs were also present in 12.8% of the adverse drug reactions reported to the Portuguese pharmacovigilance system [28]. Concerning Portuguese institutionalized older adults, one study discovered that 79.3% took PIMs according to the 2019 Beers criteria, and a positive association between polypharmacy and PIM was found (*p* < 0.001) [29]. Regarding nursing home residents, PIM was observed in 86.4% [30]. Recently, one study also found that the number of PIMs at discharge was higher than at admission in Portuguese geriatric inpatients of an internal medicine service [31].

Although some studies have been carried out in Portugal that show a high percentage of PIMs, to our knowledge, there are no studies comparing the most used criteria in primary health care. So, the aim of this study was to identify the prevalence of PIM in older adults in primary care health of the center region of Portugal, according to the 2019 Beers criteria, STOPP v2 criteria, and the Portuguese EU(7)-PIM list, and to analyze the concordance between these criteria.

## Methods

### Study design and study population

A retrospective study was performed to characterize the PIM profile among Portuguese older adults (≥ 65 years old), users of primary health care at the Regional Health Administration (Administração Regional de Saúde (ARS)) of Centro (ARSC) of Portugal. This study is part of a project that obtained ethics approval from ARSC (P33-2021). The ARSC has 488,824 older adult users enrolled in their primary health centers. The sample size included 1200 randomly selected older adults actively enrolled in ARSC primary health care facilities in the last month of the period under review, aged 65 or over in the last month of the period under review, and who had at least two primary care consultations in the period under review. This study followed the STROBE checklist for cross-sectional studies (Table S1) [32].

### Data source

Data were provided by the Shared Services of the Health Ministry (Serviços Partilhados do Ministério da Saúde) concerning the period between April 2021 and August 2022 and included the following: sex (female or male), age, health problems (according to the International Classification of Primary Care, 2nd edition – ICPC2), clinical laboratory test results, dispensed medicines, dosage, and dosage form.

### Data collection

All medicines were analyzed by a Pharmacy PhD student (DAR) who applied three criteria (Beers 2019, STOPP v2, and the Portuguese EU(7)-PIM list) for PIM identification. The classification was revised by two pharmacologists.

Three tools were used for PIM identification: (a) the Portuguese version of the EU(7)-PIM list [19], (b) the 2019 Beers criteria, and [12] (c) the STOPP v2 criteria [15]. Given the information available, it was not possible to apply all criteria. Concerning the EU(7)-PIM list, were excluded from the analysis drugs in which the classification as PIM is (i) duration of treatment-dependent (*n* = 2), (ii) therapeutic scheme-dependent (*n* = 1), (iii) duration of treatment and dose-dependent (*n* = 3), and (iv) posology-dependent (*n* = 5) (Table S2). Regarding Beers criteria, drugs whose PIM classification required information on gastroparesis (*n* = 1), first-line therapy (*n* = 1), and clinical indication (*n* = 3) were excluded from the analysis since this information was not available (Table S3). At least, according to STOPP criteria, drugs whose PIM classification required information on treatment (*n* = 3), clinical indication (*n* = 3), duration of treatment (*n* = 1), normal systolic ventricular function (*n* = 1), New York Heart Association (NYHA) Class III or IV heart failure (*n* = 1), first-line therapy (*n* = 5), contraindication or clear intolerance to other drugs (*n* = 2), concurrent significant bleeding risk (*n* = 1), coronary stent(s) inserted in the previous 12 months (*n* = 1), first deep venous thrombosis (*n* = 1), first pulmonary embolus (*n* = 1), glaucoma (*n* = 3), (m) sleep disorder (*n* = 1), posology (*n* = 1), acute or chronic respiratory failure (*n* = 1), trying other drugs before (*n* = 2), and intact uterus (*n* = 1) were excluded from the analysis since this information was not available (Table S4).

### Statistical analysis

All medicines were coded using the Anatomical and Therapeutic Classification (ATC) system. The results were presented as frequencies and percentages for categorical variables and as means (SD) for numerical variables. Statistical and descriptive analysis was conducted using the Statistical Package for Social Sciences (IBM® SPSS® Statistics version 25). PIM prevalence was defined as the number of older adults taking at least one PIM. A comparative analysis was performed between the three PIM identification criteria, and the concordance was determined according to the Lin concordance correlation coefficient. The findings were represented using their corresponding 95% confidence intervals (CIs).

## Results

### Study population characteristics

Descriptive statistics of older adults’ characteristics are described in Table 1. From the 1200 older adults included in this study, 57.6% were females, and the mean age was 76.3 (SD 7.7) years old. Within the 7921 dispensed drugs, the 2742 ICPC2 symptoms and/or complaints and the 11635 ICPC2 diagnoses and diseases, 426, 228, and 298, were different from each other, respectively. The mean number of medicines per older adult was 6.6 drugs (SD 4.2). Atorvastatin (31.4%) was the most dispensed drug followed by paracetamol (20.5%), pantoprazole (16.4%), furosemide (15.1%), and simvastatin (14.3%). The mean number of symptoms and/or complaints per older adult was 2.3 (SD 3.0). The most common observed were sleep disturbance (11.8%), low back symptom/complaint (9.3%), knee symptom/complaint (6.5%), tobacco abuse (5.9%), vertigo/dizziness (5.5%), incontinence urine (5.5%), feeling anxious/nervous/tense (5.4%), and cough (5.4%). The mean number of diagnoses and diseases per older adult was 9.7 (SD 5.6). Lipid disorder (62.8%) was the most common condition affecting older adults followed by hypertension uncomplicated (57.8%), overweight (39.7%), back syndrome with radiating pain (26.1%), diabetes non-insulin dependent (25.6%), obesity (24.8%), osteoarthrosis of the knee (23.5%), and depressive disorder (20.3%).

**Table 1:** Descriptive statistics of older adults’ characteristics (*n* = 1200)

**Sex**		***n* = 1200**
Male		509 (42.4%)
Female		691 (57.6%)
**Age (years), mean (SD)**		76.3 (SD 7.7)
**Dispensed medicines**		
Total number		7921
Mean (SD)		6.6 (SD 4.2)
Range (minimum and maximum)		0–25
**ATC code of the 5 most dispensed medicines (5th level, chemical substance)**		
C10AA05	Atorvastatin	377 (31.4%)
N02BE01	Paracetamol	246 (20.5%)
A02BC02	Pantoprazole	197 (16.4%)
C03CA01	Furosemide	181 (15.1%)
C10AA01	Simvastatin	172 (14.3%)
**ICPC2 symptoms/complaints**		
Total number		2742
Mean (SD)		2.3 (SD 3.0)
Range (minimum and maximum)		0–24
**Most observed ICPC2 symptoms/complaints**		
P06	Sleep disturbance	142 (11.8%)
L03	Low back symptom/complaint	112 (9.3%)
L15	Knee symptom/complaint	78 (6.5%)
P17	Tobacco abuse	71 (5.9%)
N17	Vertigo/dizziness	66 (5.5%)
U04	Incontinence urine	66 (5.5%)
P01	Feeling anxious/nervous/tense	65 (5.4%)
R05	Cough	65 (5.4%)
**ICPC2 diagnoses and diseases**		
Total number		11,635
Mean (SD)		9.7 (SD 5.6)
Range (minimum and maximum)		0 35
**Most observed ICPC2 diagnoses and diseases**		
T93	Lipid disorder	753 (62.8%)
K86	Hypertension uncomplicated	693 (57.8%)
T83	Overweight	476 (39.7%)
L86	Back syndrome with radiating pain	313 (26.1%)
T90	Diabetes non-insulin-dependent	307 (25.6%)
T82	Obesity	298 (24.8%)
L90	Osteoarthrosis of knee	282 (23.5%)
P76	Depressive disorder	244 (20.3%)

*ATC* Anatomical Therapeutic Chemical (ATC) Classification, *ICPC2* International Classification of Primary Care, 2nd edition, *SD* standard deviation

### PIM prevalence and frequency according to the EU(7)-PIM list, Beers criteria, and STOPP criteria

According to the Portuguese EU(7)-PIM list, 1467 PIMs were detected, 63.8% (95% CI 61.0–66.6%) of the participants took at least one PIM, and the mean number of PIM per older adult was 1.2 (SD 1.3) (Table 2). Overall, the most consumed PIMs were proton pump inhibitors (PPIs) (30.1%, 95% CI 27.6–32.9%), alprazolam (10.1%, 95% CI 8.4–11.9%), and diazepam (5.7%, 95%CI 4.4–7.1%) (Tables 3 and S5).

**Table 2:** PIMs identified according to the EU(7)-PIM list, Beers 2019, and STOPP v2 criteria

	**EU(7)-PIM list**			**Beers 2019**			**STOPP v2**		
No. of PIMs*	1467			1824			980		
**Mean PIM/older adult (SD)**	1.2 (SD 1.3)			1.5 (SD 1.5)			0.8 (SD 1.1)		

	***n***	**%**	**95% CI**	***n***	**%**	**95% CI**	***n***	**%**	**95% CI**
**No. of older adults with at least 1 PIM**	766	63.8%	61.1–66.6%	802	66.8%	64.1–69.5%	601	50.1%	47.2–53.0%
**No. of older adults with 1 PIM**	369	30.8%	28.2–33.5%	298	24.8%	22.4–27.4%	355	29.6%	27.0–32.2%
**No. of older adults with 2 PIMs**	208	17.3%	15.2–19.6%	219	18.3%	16.1–20.6%	152	12.7%,	10.8–14.7%
**No. of older adults with 3 PIMs**	112	9.3%	7.8–11.1%	139	11.6%	9.8–13.5%	69	5.8%	4.5–7.2%
**No. of older adults with 4 PIMs**	47	3.9%	2.9–5.2%	85	7.1%	5.7–8.7%	15	1.2%	0.7–2.1%
**No. of older adults with ≥ 5 PIMs**	30	2.5%	1.7–3.6%	61	5.1%	3.9–6.5%	10	0.8%	0.4–1.5%

*CI* confidence interval, *PIM* potentially inappropriate medications

Asterisk (*) symbol means an older adult can have more than one PIM

**Table 3:** The three most consumed PIMs according to the EU(7)-PIM list, Beers 2019, and STOPP v2 criteria

**Position**	**EU(7)-PIM list**			**Beers 2019**			**STOPP v2**		
	**PIM**	***n***	**% PIM**	**PIM**	***n***	**% PIM**	**PIM**	***n***	**% PIM**
1	Proton pump inhibitors	362	a) 24.7% b) 30.1% (95% CI 27.6–32.9%)	Proton pump inhibitors	362	a) 19.9% b) 30.1% (95% CI 27.6–32.9%)	Alprazolam	121	a) 12.3% b) 10.1% (95% CI 8.4–11.9%)
2	Alprazolam	121	a) 8.3% b) 10.1% (95% CI 8.4–11.9%)	Furosemide	181	a) 9.9% b) 15.1% (95% CI 13.1–17.2%)	Tramadol and paracetamol	94	a) 8.8% b) 7.8% (95% CI 6.4–9.5%)
3	Diazepam	68	a) 4.6% b) 5.7% (95% CI 4.4–7.1%)	Alprazolam	121	a) 6.6% b) 10.1% (95% CI 8.4–11.9%)	Lorazepam	73	a) 6.9% b) 6.1% (95% CI 4.8–7.6%)

*CI* confidence interval, *PIM* potentially inappropriate medications

a) percentage of PIMs per tool, b) percentage of PIMs per older adult (*n* = 1200)

Concerning Beers criteria, we have identified a total of 1824 PIMs, with 66.8% (95% CI 64.1–69.5%) of the older adults taking at least one PIM and with a mean number of PIM per older adult of 1.5 (SD 1.5) (Table 2). The most consumed PIMs were PPIs (30.1%, 95% CI 27.6–32.9%), furosemide (15.1%, 95% CI 13.1–17.2%), and alprazolam (10.1%, 95% CI 8.4–11.9%) (Tables 3 and S6). Through the application of Table 2 of Beers 2019 criteria (medications that are potentially inappropriate in most older adults), 916 PIMs were detected, with PPIs with the highest value of PIM frequency (39.5%), followed by alprazolam (13.2%) and diazepam (7.4%) (Table S7). Regarding Table 3 of Beers 2019 criteria (medications that are potentially inappropriate in older adults with certain conditions), 157 PIMs were identified (Table S8). The application of Table 4 of Beers 2019 criteria resulted in 856 medications that should be used with caution in older adults, with furosemide presenting the highest value of PIM frequency (21.1%) (Table S9). According to Table 5 of Beers 2019 criteria (potentially clinically important drug-drug interactions that should be avoided in older adults), 206 potentially drug-drug interactions were identified (Table S10). Any combination of three or more of central nervous system-active drugs had the highest interaction frequency (52.9%). Concerning Table 6 of Beers criteria, 17 medications that should be avoided or have their dosage reduced with varying levels of kidney function in older adults were identified, being edoxaban the most frequent (41.2%) (Table S11). The frequency of drugs with strong anticholinergic properties through the application of Table 7 of Beers 2019 criteria was 154, with cyclobenzaprine being the most frequent (26.0%) (Table S12).

**Table 4:** PIM frequency according to the anatomical group

**ATC code (1st level, anatomical main group)**	**Dispensed medicines**	**EU(7)-PIM list (%)**	**Beers 2019 (%)**	**STOPP v2 (%)**
Alimentary tract and metabolism (A)	1231	420 (34.1%)	369 (30.0%)	18 (1.5%)
Blood and blood-forming organs (B)	396	140 (35.3%)	95 (24.0%)	14 (3.5%)
Cardiovascular system (C)	2482	121 (4.9%)	299 (12.1%)	74 (3.0%)
Dermatologicals (D)	129	0	0	0
Genito urinary system and sex hormones (G)	269	35 (13.0%)	35 (13.0%)	9 (3.4%)
Systemic hormonal preparations, excl. Sex hormones and insulins (H)	172	0	1 (0.6%)	3 (1.7%)
Antiinfectives for systemic use (J)	394	2 (0.5%)	1 (0.3%)	0
Antineoplastic and immunomodulating agents (L)	8	0	0	0
Musculo-skeletal system (M)	619	263 (42.5%)	167 (27.0%)	240 (38.8%)
Nervous system (N)	1762	479 (27.2%)	857 (48.6%)	622 (35.3%)
Antiparasitic products, insecticides, and repellents (P)	8	0	0	0
Respiratory system (R)	314	7 (2.2%)	0	0
Sensory organs (S)	137	0	0	0
Various (V)	0	0	0	0

**Table 5:** LIN concordance correlation coefficient

**PIM criteria**	**CCC (95% CI)**
EU(7)-PIM list *vs.* Beers	0.833 (0.648–0.925)
EU(7)-PIM list *vs.* STOPP	0.735 (0.366–0.904)
Beers *vs.* STOPP	0.800 (0.568–0.914)

For STOPP criteria, a total of 980 PIM was obtained, 50.1% (95% CI 47.2–53.0%) of the older adults take at least one PIM and the mean number of PIM per older adult was 0.8 (SD 1.1) (Table 2). According to these criteria, alprazolam was the most consumed PIM (10.1%, 95% CI 8.4–11.9%), followed by tramadol and paracetamol (7.8%, 7.8%, 95% CI 6.4–9.5%) and lorazepam (6.1%, 6.1%, 95% CI 4.8–7.6%) (Tables 3 and S12). Besides, 85 duplicate drug classes were identified according to Section A.3 of the STOPP criteria.

### PIM frequency according to the anatomical group and concordance between criteria

**Fig. 1:**
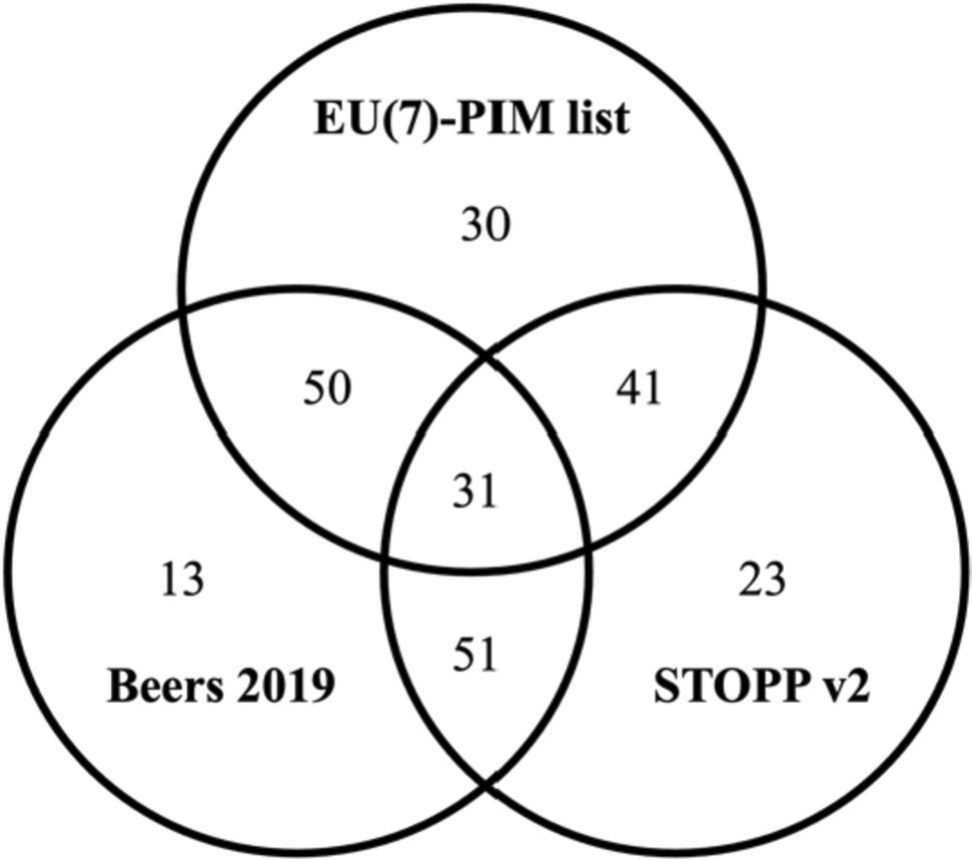
PIM number according to the EU(7)-PIM list, Beers criteria, and STOPP criteria

According to Table 4, drugs that act on the nervous system were the most identified as PIM for all the three criteria. However, some differences were found regarding PIM frequency according to the anatomical group. From the 1231 dispensed medicines belonging to the alimentary tract and metabolism group, 34.1% and 30.0% were considered PIM according to the EU(7)-PIM list and Beers criteria, but only 1.5% by the STOPP criteria. Major differences can also be found in the dispensed drugs belonging to the blood and blood-forming organs group, with 35.5% and 24.0% considered PIM according to the EU(7)-PIM list and Beers criteria, but only 3.5% by the STOPP criteria. Concerning the cardiovascular system, the major difference was found in the Beers criteria which considered 12.1% of the drugs dispensed as PIM, unlike the EU(7)-PIM list (4.9%) and STOPP criteria (3.0%). For the systemic hormonal preparations (excluding sex hormones and insulins) group, PIMs were found according to Beers (0.6%) and STOPP criteria (1.7%). Otherwise, drugs belonging to the antiinfectives for systemic use group were identified as PIM by the EU(7)-PIM list (0.5%) and Beers criteria (0.3%). Regarding the respiratory system, drugs were considered PIM only by the EU(7)-PIM list (2.2%). A poor concordance between criteria was found according to Lin’s concordance correlation coefficient (Table 5). The highest concordance coefficient was found between the EU(7)-PIM list and the Beers criteria, and the lowest between the EU(7)-PIM list and STOPP criteria. The three criteria have in common 31 unique PIMs (Fig. 1). The EU(7)-PIM list had 50 PIMs in common with the Beers criteria and 41 with the STOPP criteria. Beers and STOPP criteria shared 51 PIMs.

## Discussion

This is the first study, to our knowledge, comparing PIM prevalence according to the three different criteria applied simultaneously to the same sample of older adults in primary care in Portugal. PIM prevalence was high, but it was different according to each criterion applied. According to our results, the 2019 Beers criteria are the most sensitive tool to detect PIMs when compared to the EU(7)-PIM list and the STOPP v2 criteria (66.8%, 95% CI 64.1–69.5% vs. 63.8%, 95% CI 61.1–66.6% vs. 50.1%, 95% CI 47.2–53.0%, respectively). A poor concordance between criteria was found which means there was minimal overlap between the criteria.

Several studies have also shown a high PIM prevalence in older adult primary care users, ranging from 23.6 to 75.9% [33–39]. The Beers criteria are usually the tool that identifies most PIMs in older population when several screening tools are compared to each other [33, 36–38]. Perhaps because this tool considers a drug as PIM-based (i) in the older adult medication profile, (ii) in their diseases or syndromes, and (iii) in their levels of kidney function [12]. Besides, the criteria also contain a list of drugs for which there is some cause of concern but for which the evidence is yet insufficient [12]. The STOPP criteria identified the lowest number of PIMs, maybe because for this tool to be applied, many patients’ clinical information is required, and that information was not available equally in all settings [40]. In fact, according to one study performed in nursing homes, sufficient information was available for only 32.5% of the potential STOPP criteria situations [40].

The high mean number of medicines per older adult (6.6, SD 4.2) and the high PIM prevalence presented in this study suggest that polypharmacy can be an important predictor for PIM occurrence, as identified in previous studies [41–43]. According to a recent systematic review, the prevalence of polypharmacy among older adults is 46.0% [44]. Additional reasons may include the presence of multiple comorbidities [45], with our results also revealing a high mean number of conditions per older adult (9.7, SD 5.6). Like our study, hypertension and dyslipidemia are among the most common conditions found in older adults [46].

Different PIMs were identified by the criteria applied. As in our study, PPIs are often recognized as one of the most inappropriately consumed drug classes [47, 48]. They have the indication to treat gastroesophageal reflux disease (GERD), Barrett’s esophagus/intestinal metaplasia, functional dyspepsia, *Helicobacter pylori* eradication, and pathological hypersecretory conditions such as Zollinger–Ellison syndrome [49, 50]. However, none of these conditions was identified as one of the most prevalent in our sample, which highlights the potential inappropriate consumption of PPIs in the older population. In addition, long-term use of PPIs is very frequent in older adults [51] and has been associated with several adverse effects, such as dementia; osteoporosis; increased risk of micronutrient deficiencies (calcium, iron, magnesium, vitamin B12); increased risk of *Clostridium difficile*, *Campylobacter*, *Salmonella*, and community-acquired pneumonia infection; kidney disease; and myocardial infarction [52].

Alprazolam was the only common top-dispensed PIM for all criteria. Consistently with several studies, benzodiazepines are one of the most common PIMs identified [53] and widely used by Portuguese older adults. In fact, some studies conclude that alprazolam was the most found PIM in Portuguese samples of institutionalized older adults [29, 54]. The high consumption of benzodiazepines may be related with sleep disturbance and feelings of anxiety and nervousness which were identified as two of the most prevalent symptoms/complaints in our sample (11.8% and 5.4%, respectively). In fact, anxiety disorders are one of the most prevalent mental health illnesses in the older population [55].

Central nervous system-active drugs had the highest interaction frequency (52.9%), regarding potentially drug-drug interactions identified through the application of Table 5 of Beers criteria. This result is in line with an international consensus list of potentially clinically significant drug-drug interactions in older people [56]. Besides, drugs acting in the central nervous system are commonly implicated in potentially serious interactions over time [57].

Considering medications that should be avoided or have their dosage reduced according to the levels of kidney function in older adults through the application of Table 6 of Beers criteria, edoxaban had the higher PIM frequency (41.2%). Direct Oral Anticoagulants (DOACs), such as edoxaban, significantly reduce the occurrence of stroke and embolism in older adults with atrial fibrillation [58], being a more effective and safer alternative to warfarin [59], possibly explaining their high consumption among this population. However, according to Beers 2019 criteria, there is a lack of evidence of efficacy or safety in patients with a CrCl < 30 mL/min, so dose reduction is advised if CrCl 15–50 mL/min and should be avoided if CrCl < 15 or > 95 mL/min [12]. Besides, there is less advantage of DOACs over vitamin K antagonists (VKAs), the more the renal function declines [60].

Cyclobenzaprine, a muscle relaxant, was the most identified drug with strong anticholinergic properties (26.0%) in our study through the application of Table 7 of Beers criteria. In other studies, cyclobenzaprine was also among the most found potentially inappropriate anticholinergic medications in older adults [61, 62]. Sometimes, muscle relaxants are inappropriately used as an alternative to conventional pain medications by older adults [63], and cyclobenzaprine was associated with an increased risk of injury in older adults [64].

The poor concordance between criteria could be related to the applicability of different requirements of each screening tool and the different information available in each setting. The EU(7)-PIM list and the STOPP criteria presented the lowest concordance value, maybe because the EU(7)-PIM list classification as PIM only considers the current medication profile of older adults, including dose and duration of treatment [17], while STOPP criteria also considers previous medication, current and past medical conditions, and laboratory data [15, 40]. In Portugal, one study also compared the same criteria but in older inpatients of an internal medicine ward, with the Beers criteria also identifying the highest number of patients with at least one PIM (92.0%), although with a lower concordance between criteria (< 63.4%) [65]. A previous study in Brazil found high concordance among the 2015 Beers criteria, STOPP v2, the EU(7)-PIM list, and Taiwan criteria [37]. However, the 2019 update made to the 2015 Beers criteria includes the removal of 2 medications, the addition of 3, and the modification to the recommendations related to 6 medications/medication classes [11, 12], probably decreasing concordance with other criteria.

This study contributes with valuable data on the most prevalent PIMs and the concordance between different criteria, which will be useful for the development of interventions designed to improve PIM prescription. The main limitations of this study are the reasons why older adults are taking these drugs are not known, and the exclusion of some criteria of the three screening tools that could not be applied may have underestimated the PIM number.

## Conclusion

Our study reveals high PIM prevalence in Portuguese primary health care older adults, with varying prevalence rates according to different criteria, emphasizing the need for targeted interventions and improved prescribing practices. Future studies should focus on the occurrence of negative outcomes in the older adults associated with PIM consumption, and more interventions are needed to reduce PIM use in this population.

### Supplementary Information

Below is the link to the electronic supplementary material.

Supplementary file1 (DOCX 95 KB)

## Data Availability

The data that support the findings of this study are available from the corresponding author upon reasonable request.
