# Supplementary material for: Comparing AGS Beers 2019, STOPP version 2, and EU(7)-PIM list in Portuguese older adults in primary health care
Source: Eur J Clin Pharmacol. 2024 Feb 6;80(4):603–12. doi: 10.1007/s00228-024-03633-5 (PMC10937751; doi:10.1007/s00228-024-03633-5)
Supplement: Supplementary file 1 — Supplementary file1 (DOCX 95 KB) [file 228_2024_3633_MOESM1_ESM.docx]

**Supplementary Material**

**Table S1:** STROBE Statement—Checklist of items that should be included in reports of cross-sectional studies.

|  | Item No | Recommendation |
| --- | --- | --- |
| **Title and abstract** | 1 | (*a*) Indicate the study’s design with a commonly used term in the title or the abstract |
|  |  | (*b*) Provide in the abstract an informative and balanced summary of what was done and what was found |
| Introduction | | |
| Background/rationale | 2 | Explain the scientific background and rationale for the investigation being reported |
| Objectives | 3 | State specific objectives, including any prespecified hypotheses |
| Methods | | |
| Study design | 4 | Present key elements of study design early in the paper |
| Setting | 5 | Describe the setting, locations, and relevant dates, including periods of recruitment, exposure, follow-up, and data collection |
| Participants | 6 | (*a*) Give the eligibility criteria, and the sources and methods of selection of participants |
| Variables | 7 | Clearly define all outcomes, exposures, predictors, potential confounders, and effect modifiers. Give diagnostic criteria, if applicable |
| Data sources/ measurement | 8* | For each variable of interest, give sources of data and details of methods of assessment (measurement). Describe comparability of assessment methods if there is more than one group |
| Bias | 9 | Describe any efforts to address potential sources of bias |
| Study size | 10 | Explain how the study size was arrived at |
| Quantitative variables | 11 | Explain how quantitative variables were handled in the analyses. If applicable, describe which groupings were chosen and why |
| Statistical methods | 12 | (*a*) Describe all statistical methods, including those used to control for confounding |
|  |  | (*b*) Describe any methods used to examine subgroups and interactions |
|  |  | (*c*) Explain how missing data were addressed |
|  |  | (*d*) If applicable, describe analytical methods taking account of sampling strategy |
|  |  | (*e*) Describe any sensitivity analyses |
| Results | | |
| Participants | 13* | (a) Report numbers of individuals at each stage of study—eg numbers potentially eligible, examined for eligibility, confirmed eligible, included in the study, completing follow-up, and analysed |
|  |  | (b) Give reasons for non-participation at each stage |
|  |  | (c) Consider use of a flow diagram |
| Descriptive data | 14* | (a) Give characteristics of study participants (eg demographic, clinical, social) and information on exposures and potential confounders |
|  |  | (b) Indicate number of participants with missing data for each variable of interest |
| Outcome data | 15* | Report numbers of outcome events or summary measures |
| Main results | 16 | (*a*) Give unadjusted estimates and, if applicable, confounder-adjusted estimates and their precision (eg, 95% confidence interval). Make clear which confounders were adjusted for and why they were included |
|  |  | (*b*) Report category boundaries when continuous variables were categorized |
|  |  | (*c*) If relevant, consider translating estimates of relative risk into absolute risk for a meaningful time period |
| Other analyses | 17 | Report other analyses done—eg analyses of subgroups and interactions, and sensitivity analyses |
| Discussion | | |
| Key results | 18 | Summarise key results with reference to study objectives |
| Limitations | 19 | Discuss limitations of the study, taking into account sources of potential bias or imprecision. Discuss both direction and magnitude of any potential bias |
| Interpretation | 20 | Give a cautious overall interpretation of results considering objectives, limitations, multiplicity of analyses, results from similar studies, and other relevant evidence |
| Generalisability | 21 | Discuss the generalisability (external validity) of the study results |
| Other information | | |
| Funding | 22 | Give the source of funding and the role of the funders for the present study and, if applicable, for the original study on which the present article is based |

*Give information separately for exposed and unexposed groups.

**Note:** An Explanation and Elaboration article discusses each checklist item and gives methodological background and published examples of transparent reporting. The STROBE checklist is best used in conjunction with this article (freely available on the Web sites of PLoS Medicine at http://www.plosmedicine.org/, Annals of Internal Medicine at http://www.annals.org/, and Epidemiology at http://www.epidem.com/). Information on the STROBE Initiative is available at www.strobe-statement.org.

**Table S2:** EU(7)-PIM excluded PIM with reasons (n=11).

| ATC code | EU(7)-PIM | Reason of exclusion |
| --- | --- | --- |
| A07DA03 | Loperamide (>2 days) | (a) the classification as PIM is duration of treatment-dependent |
| no ATC, treatment concept PIM | Insulin, sliding scale | (b) the classification of PIM is therapeutic scheme-dependent |
| J01XE01 | Nitrofurantoin (>1 week) | (a) the classification as PIM is duration of treatment-dependent |
| M01AE01 | Ibuprofen (>3 x 400 mg/d or for a period longer than one week) | (c) the classification as PIM is duration of treatment and dose-dependent |
| M01AE02 | Naproxen (>2 x 250 mg/d or for a period longer than one week) | (c) the classification as PIM is duration of treatment and dose-dependent |
| N05AD01 | Haloperidol (>2 mg single dose; >5mg/d) | (c) the classification as PIM is duration of treatment and dose-dependent |
| N05AH03 | Olanzapine (>10 mg/d) | (d) the classification as PIM is posology-dependent |
| N05BA04 | Oxazepam (>60 mg/d) | (d) the classification as PIM is posology-dependent |
| N05BA06 | Lorazepam (>1 mg/d) | (d) the classification as PIM is posology-dependent |
| N05CD09 | Brotizolam (> 0,125 mg/d) | (d) the classification as PIM is posology-dependent |
| N05CF02 | Zolpidem (> 5mg/d) | (d) the classification as PIM is posology-dependent |

**Table S3:** Beers 2019 criteria excluded with reasons (n=5).

| Table 2. 2019 American Geriatrics Society Beers Criteria® for Potentially Inappropriate Medication Use in Older Adults | | |
| --- | --- | --- |
| Organ System, Therapeutic Category, Drug(s) | **Criteria - Recommendation** | **Reason of exclusion** |
| Metoclopramide | Avoid, unless for gastroparesis with duration of use not to exceed 12 weeks except in rare cases | Information about gastroparesis not available |
| Digoxin for first-line treatment of atrial fibrillation or of heart failure | Avoid this rate control agent as first-line therapy for atrial fibrillation.  Avoid as first-line therapy for heart failure. | Information about first-line therapy not available |
| Peripheral alpha-1 blockers for treatment of hypertension  Doxazosin  Prazosin  Terazosin | Avoid use as an antihypertensive | Information about clinical indication not available |
| Desmopressin | Avoid for treatment of nocturia or nocturnal polyuria | Information about clinical indication not available |
| Nitrofurantoin | Avoid in individuals with creatinine clearance <30 mL/min or for long-term suppression | Information about clinical indication not available |

**Table S4:** STOPP v2 criteria excluded with reasons (n=28).

| Section A: Indication of medication | | Reason of exclusion |
| --- | --- | --- |
| 1 | Any drug prescribed without an evidence-based clinical indication. | Information about clinical indication not available |
| 2 | Any drug prescribed beyond the recommended duration, where treatment duration is well defined. | Information about duration of treatment not available |
| Section B: Cardiovascular System | | **Reason of exclusion** |
| 1 | Digoxin for heart failure with normal systolic ventricular function (no clear evidence of benefit) | Information about normal systolic ventricular function not available |
| 2 | Verapamil or diltiazem with NYHA Class III or IV heart failure (may worsen heart failure). | Information about NYHA Class III or IV heart failure not available |
| 5 | Amiodarone as first-line antiarrhythmic therapy in supraventricular tachyarrhythmias (higher risk of side-effects than beta-blockers, digoxin, verapamil or diltiazem). | Information about first-line therapy not available |
| 6 | Loop diuretic as first-line treatment for hypertension (safer, more effective alternatives available). | Information about first-line therapy not available |
| 10 | Centrally-acting antihypertensives (e.g. methyldopa, clonidine, moxonidine, rilmenidine, guanfacine), unless clear intolerance of, or lack of efficacy with, other classes of antihypertensives (centrally-active antihypertensives are generally less well tolerated by older people than younger people). | Information about clear intolerance of, or lack of efficacy with, other classes of antihypertensives not available |
| Section C: Antiplatelet/Anticoagulant Drugs | | **Reason of exclusion** |
| 3 | Aspirin, clopidogrel, dipyridamole, vitamin K antagonists, direct thrombin inhibitors or factor Xa inhibitors with concurrent significant bleeding risk, i.e. uncontrolled severe hypertension, bleeding diathesis, recent non-trivial spontaneous bleeding) (high risk of bleeding). | Information about concurrent significant bleeding risk not available |
| 4 | Aspirin plus clopidogrel as secondary stroke prevention, unless the patient has a coronary stent(s) inserted in the previous 12 months or concurrent acute coronary syndrome or has a high grade symptomatic carotid arterial stenosis (no evidence of added benefit over clopidogrel monotherapy). | Information about coronary stent(s) inserted in the previous 12 months not available |
| 8 | Vitamin K antagonist, direct thrombin inhibitor or factor Xa inhibitors for first deep venous thrombosis without continuing provoking risk factors (e.g. thrombophilia) for > 6 months, (no proven added benefit). | Information about first deep venous thrombosis not available |
| 9 | Vitamin K antagonist, direct thrombin inhibitor or factor Xa inhibitors for first pulmonary embolus without continuing provoking risk factors (e.g. thrombophilia) for > 12 months (no proven added benefit). | Information about first pulmonary embolus not available |
| Section D: Central Nervous System and Psychotropic Drugs | | **Reason of exclusion** |
| 1 | TriCyclic Antidepressants (TCAs) with dementia, narrow angle glaucoma, cardiac conduction abnormalities, prostatism, or prior history of urinary retention (risk of worsening these conditions). | Information about glaucoma not available |
| 2 | Initiation of TriCyclic Antidepressants (TCAs) as first-line antidepressant treatment (higher risk of adverse drug reactions with TCAs than with SSRIs or SNRIs). | Information about first-line therapy not available |
| 7 | Anticholinergics/antimuscarinics to treat extra-pyramidal side-effects of neuroleptic medications (risk of anticholinergic toxicity). | Information about treatment for extra-pyramidal side-effects of neuroleptic medications not available |
| 9 | Neuroleptic antipsychotic in patients with behavioural and psychological symptoms of dementia (BPSD) unless symptoms are severe and other non-pharmacological treatments have failed (increased risk of stroke). | Information about other non-pharmacological treatments failure not available |
| 10 | Neuroleptics as hypnotics, unless sleep disorder is due to psychosis or dementia (risk of confusion, hypotension, extra-pyramidal side effects, falls). | Information about sleep disorder not available |
| 12 | Phenothiazines as first-line treatment, since safer and more efficacious alternatives exist (phenothiazines are sedative, have significant anti-muscarinic toxicity in older people, with the exception of prochlorperazine for nausea/vomiting/vertigo, chlorpromazine for relief of persistent hiccoughs and levomepromazine as an anti-emetic in palliative care). | Information about first-line therapy not available |
| Section E: Renal System. The following drugs are potentially inappropriate in older people with acute or chronic kidney disease with renal function below particular levels of eGFR (refer to summary of product characteristics datasheets and local formulary guidelines) | | **Reason of exclusion** |
| 1 | Digoxin at a long-term dose greater than 125μg/day if eGFR < 30 ml/min/1.73m2 (risk of digoxin toxicity if plasma levels not measured). | Information about specific dosage for each patient not available |
| Section G: Respiratory System | | **Reason of exclusion** |
| 3 | Anti-muscarinic bronchodilators (e.g. ipratropium, tiotropium) with a history of narrow angle glaucoma (may exacerbate glaucoma) or bladder outflow obstruction (may cause urinary retention). | Information about glaucoma not available |
| 4 | Benzodiazepines with acute or chronic respiratory failure i.e. pO2 < 8.0 kPa ± pCO2 > 6.5 kPa (risk of exacerbation of respiratory failure). | Information about acute or chronic respiratory failure not available |
| Section H: Musculoskeletal System | | **Reason of exclusion** |
| 3 | Long-term use of NSAID (>3 months) for symptom relief of osteoarthritis pain where paracetamol has not been tried (simple analgesics preferable and usually as effective for pain relief). | Information about trying paracetamol before not available |
| 4 | Long-term corticosteroids (>3 months) as monotherapy for rheumatoid arthrtitis (risk of systemic corticosteroid side-effects). | Information about monotherapy for rheumatoid arthrtitis not available |
| 5 | Corticosteroids (other than periodic intra-articular injections for mono-articular pain) for osteoarthritis (risk of systemic corticosteroid side-effects). | Information about intra-articular injections for mono-articular pain not available |
| 6 | Long-term NSAID or colchicine (>3 months) for chronic treatment of gout where there is no contraindication to a xanthine-oxidase inhibitor (e.g. allopurinol, febuxostat) (xanthine-oxidase inhibitors are first choice prophylactic drugs in gout). | Information about contraindication to a xanthine-oxidase inhibitor not available |
| Section I: Urogenital System | | **Reason of exclusion** |
| 1 | Antimuscarinic drugs with dementia, or chronic cognitive impairment (risk of increased confusion, agitation) or narrow-angle glaucoma (risk of acute exacerbation of glaucoma), or chronic prostatism (risk of urinary retention). | Information about glaucoma not available |
| Section J. Endocrine System | | **Reason of exclusion** |
| 5 | Oral oestrogens without progestogen in patients with intact uterus (risk of endometrial cancer). | Information about intact uterus not available |
| Section L: Analgesic Drugs | | **Reason of exclusion** |
| 1 | Use of oral or transdermal strong opioids (morphine, oxycodone, fentanyl, buprenorphine, diamorphine, methadone, tramadol, pethidine, pentazocine) as first line therapy for mild pain (WHO analgesic ladder not observed). | Information about first-line therapy not available |
| 3 | Long-acting opioids without short-acting opioids for break-through pain (risk of persistence of severe pain). | Information about trying short-acting opioids before not available |

**Table S5:** Total number of PIM dispensed, according to the EU(7)-PIM list.

| ATC code | PIM EU(7)-PIM list | PIM frequency  n=1467 | PIM % |
| --- | --- | --- | --- |
| A02BC | Proton pump inhibitors (PPI) (>8 weeks) | 362 | 24.7% |
| A03FA01 | Metoclopramide | 24 | 1.6% |
| A10BB01 | Glibenclamide | 2 | 0.1% |
| A10BB12 | Glimepiride | 2 | 0.1% |
| A10BF01 | Acarbose | 3 | 0.2% |
| A10BG03 | Pioglitazone | 1 | 0.1% |
| A10BH01 | Sitagliptine | 18 | 1.2% |
| A10BH02 | Vildagliptine | 8 | 0.6% |
| B01AA07 | Acenocoumarol | 4 | 0.3% |
| B01AC05 | Ticlopidine | 8 | 0.6% |
| B01AC07 | Dipyridamole | 1 | 0.1% |
| B01AE07 | Dabigatran etexilate | 13 | 0.9% |
| B01AF01 | Rivaroxaban | 48 | 3.3% |
| B01AF02 | Apixaban | 57 | 3.9% |
| B03AA | Iron supplements / Ferrous sulfate (>325 mg/d) | 9 | 0.6% |
| C01AA05 | Digoxin | 19 | 1.3% |
| C01AA08 | Metildigoxin | 2 | 0.1% |
| C01BC03 | Propafenone | 12 | 0.8% |
| C01BD01 | Amiodarone | 14 | 1.0% |
| C01EB15 | Trimetazidine | 10 | 0.7% |
| C01EB17 | Ivabradine | 2 | 0.1% |
| C02AC06 | Rilmenidine | 7 | 0.5% |
| C02CA04 | Doxazosin | 3 | 0.2% |
| C03DA01 | Spironolactone (>25 mg/d) | 3 | 0.2% |
| C04AX17 | Vinburnine | 1 | 0.1% |
| C07AA05 | Propranolol | 20 | 1.4% |
| C07AA07 | Sotalol | 2 | 0.1% |
| C08CA05 | Nifedipine (sustained release) | 13 | 0.9% |
| C08DA01 | Verapamil | 5 | 0.3% |
| C08DB01 | Diltiazem | 8 | 0.6% |
| G04BD02 | Flavoxat | 13 | 0.9% |
| G04BD04 | Oxybutynine (non-sustained release) | 5 | 0.3% |
| G04BD08 | Solifenacin | 5 | 0.3% |
| G04BD09 | Trospium | 12 | 0.8% |
| J01MA17 | Prulifloxacin | 2 | 0.1% |
| M01AB05 | Diclofenac | 44 | 3.0% |
| M01AB08 | Etodolac | 38 | 2.6% |
| M01AB11 | Acemetacin | 58 | 4.0% |
| M01AB16 | Aceclofenac | 7 | 0.5% |
| M01AC01 | Piroxicam | 2 | 0.1% |
| M01AC06 | Meloxicam | 1 | 0.1% |
| M01AE03 | Ketoprofen | 7 | 0.5% |
| M01AH01 | Celecoxib | 9 | 0.6% |
| M01AH05 | Etoricoxib | 37 | 2.5% |
| M03BX01 | Baclofen | 1 | 0.1% |
| M03BX02 | Tizanidine | 4 | 0.3% |
| M03BX08 | Cyclobenzaprine | 40 | 2.7% |
| M04AC01 | Colchicin | 15 | 1.0% |
| N02AX02 | Tramadol (sustained release) | 16 | 1.1% |
|  | Tramadol (non-sustained release) | 4 | 0.3% |
| N02CC | Triptanes | 1 | 0.1% |
| N03AA02 | Phenobarbital | 2 | 0.1% |
| N03AB02 | Phenytoin | 1 | 0.1% |
| N03AE01 | Clonazepam | 16 | 1.1% |
| N03AF01 | Carbamazepine | 3 | 0.2% |
| N03AX11 | Topiramate | 4 | 0.3% |
| N04AA01 | Trihexyphenidyl | 1 | 0.1% |
| N04AA02 | Biperiden | 1 | 0.1% |
| N04BC04 | Ropinirole | 2 | 0.1% |
| N04BC08 | Piribedil | 2 | 0.1% |
| N04BC09 | Rotigotine | 1 | 0.1% |
| N04BD01 | Selegiline | 1 | 0.1% |
| N05AA01 | Chlorpromazine | 1 | 0.1% |
| N05AA06 | Cyamemazine | 2 | 0.1% |
| N05AN01 | Lithium | 1 | 0.1% |
| N05AX08 | Risperidone (>6 weeks) | 8 | 0.6% |
| N05AX12 | Aripiprazole | 1 | 0.1% |
| N05BA01 | Diazepam | 68 | 4.6% |
| N05BA05 | Dipotassium clorazepate | 7 | 0.5% |
| N05BA08 | Bromazepam | 32 | 2.2% |
| N05BA09 | Clobazam | 3 | 0.2% |
| N05BA12 | Alprazolam | 121 | 8.3% |
| N05BA18 | (Ethyl-) Loflazepate | 23 | 1.6% |
| N05BA22 | Cloxazolam | 9 | 0.6% |
| N05BB01 | Hydroxyzine | 18 | 1.2% |
| N05CD01 | Flurazepam | 2 | 0.1% |
| N05CD04 | Estazolam | 4 | 0.3% |
| N05CD05 | Triazolam | 1 | 0.1% |
| N05CD08 | Midazolam | 4 | 0.3% |
| N05CD11 | Loprazolam (>0.5 mg/d) | 1 | 0.1% |
| N06AA04 | Clomipramine | 9 | 0.6% |
| N06AA06 | Trimipramine | 1 | 0.1% |
| N06AA09 | Amitriptyline | 24 | 1.6% |
| N06AA16 | Dosulepin | 2 | 0.1% |
| N06AA21 | Maprotiline | 2 | 0.1% |
| N06AB03 | Fluoxetine | 36 | 2.5% |
| N06AB05 | Paroxetine | 17 | 1.2% |
| N06AB08 | Fluvoxamine | 2 | 0.1% |
| N06AX12 | Bupropion | 7 | 0.5% |
| N06AX16 | Venlafaxine | 18 | 1.2% |
| R06AX22 | Ebastine | 7 | 0.5% |

**Table S6:** Total number of PIM dispensed, according to Beers 2019 criteria (excluding Beers criteria Table 5).

| ATC code | PIM Beers 2019 criteria | PIM frequency  n=1824 | PIM % |
| --- | --- | --- | --- |
| A02BC | Proton pump inhibitors | 362 | 19.9% |
| A03FA01 | Metoclopramide | 3 | 0.2% |
| A10BB01 | Glibenclamide | 2 | 0.1% |
| A10BB12 | Glimepiride | 2 | 0.1% |
| B01AC06 | Acetylsalicylic acid | 45 | 2.5% |
| B01AC07 | Dipyridamole | 1 | 0.1% |
| B01AE07 | Dabigatran etexilate | 7 | 0.4% |
| B01AF01 | Rivaroxaban | 34 | 1.9% |
| B01AF02 | Apixaban | 1 | 0.1% |
| B01AF03 | Edoxaban | 7 | 0.4% |
| C01BD01 | Amiodarone | 6 | 0.3% |
| C03BA04 | Chlortalidone | 23 | 1.3% |
| C03BA11 | Indapamide | 52 | 2.9% |
| C03CA01 | Furosemide | 181 | 9.9% |
| C03DA01 | Spironolactone | 35 | 1.9% |
| C08DA01 | Verapamil | 1 | 0.1% |
| C08DB01 | Diltiazem | 1 | 0.1% |
| G04BD02 | Flavoxate | 13 | 0.7% |
| G04BD04 | Oxybutynin | 5 | 0.3% |
| G04BD08 | Solifenacin | 5 | 0.3% |
| G04BD09 | Trospium | 12 | 0.7% |
| H02AB13 | Deflazacort | 1 | 0.1% |
| J01EE01 | Sulfamethoxazole and trimethoprim | 1 | 0.1% |
| M01AB05 | Diclofenac | 22 | 1.2% |
| M01AB08 | Etodolac | 21 | 1.2% |
| M01AB11 | Acemetacin | 5 | 0.3% |
| M01AC01 | Piroxicam | 1 | 0.1% |
| M01AC06 | Meloxicam | 1 | 0.1% |
| M01AE01 | Ibuprofen | 23 | 1.3% |
| M01AE02 | Naproxen | 51 | 2.8% |
| M01AE03 | Ketoprofen | 2 | 0.1% |
| M01AX17 | Nimesulide | 1 | 0.1% |
| M03BX08 | Cyclobenzaprine | 40 | 2.2% |
| N02AB03 | Fentanyl | 1 | 0.1% |
| N02AJ06 | Codeine and paracetamol | 1 | 0.1% |
| N02AJ13 | Tramadol and paracetamol | 12 | 0.7% |
| N02AX02 | Tramadol | 20 | 1.1% |
| N02AX06 | Tapentadol | 1 | 0.1% |
| N03AX14 | Levetiracetam | 3 | 0.2% |
| N03AA02 | Phenobarbital | 2 | 0.1% |
| N03AE01 | Clonazepam | 16 | 0.9% |
| N03AF01 | Carbamazepine | 3 | 0.2% |
| N04AA01 | Trihexyphenidyl | 1 | 0.1% |
| N05AA01 | Chlorpromazine | 1 | 0.1% |
| N05AA06 | Cyamemazine | 2 | 0.1% |
| N05AD01 | Haloperidol | 3 | 0.2% |
| N05AD03 | Melperone | 3 | 0.2% |
| N05AH03 | Olanzapine | 8 | 0.4% |
| N05AH04 | Quetiapine | 31 | 1.7% |
| N05AL03 | Tiapride | 3 | 0.2% |
| N05AL05 | Amisulpride | 14 | 0.8% |
| N05AX08 | Risperidone | 9 | 0.5% |
| N05AX12 | Aripiprazole | 1 | 0.1% |
| N05BA01 | Diazepam | 68 | 3.7% |
| N05BA04 | Oxazepam | 16 | 0.9% |
| N05BA05 | Potassium clorazepate | 7 | 0.4% |
| N05BA06 | Lorazepam | 73 | 4.0% |
| N05BA08 | Bromazepam | 2 | 0.1% |
| N05BA12 | Alprazolam | 121 | 6.6% |
| N05BA18 | Ethyl loflazepate | 2 | 0.1% |
| N05BA22 | Cloxazolam | 2 | 0.1% |
| N05BB01 | Hydroxyzine | 18 | 1.0% |
| N05CD01 | Flurazepam | 2 | 0.1% |
| N05CD04 | Estazolam | 4 | 0.2% |
| N05CD05 | Triazolam | 1 | 0.1% |
| N05CD09 | Brotizolam | 1 | 0.1% |
| N05CF02 | Zolpidem | 19 | 1.0% |
| N06AA04 | Clomipramine | 9 | 0.5% |
| N06AA06 | Trimipramine | 1 | 0.1% |
| N06AA09 | Amitriptyline | 24 | 1.3% |
| N06AA16 | Dosulepin | 2 | 0.1% |
| N06AA21 | Maprotiline | 2 | 0.1% |
| N06AB03 | Fluoxetine | 36 | 2.0% |
| N06AB04 | Citalopram | 4 | 0.2% |
| N06AB05 | Paroxetine | 17 | 0.9% |
| N06AB06 | Sertraline | 77 | 4.2% |
| N06AB08 | Fluvoxamine | 2 | 0.1% |
| N06AB10 | Escitalopram | 71 | 3.9% |
| N06AX05 | Trazodone | 68 | 3.7% |
| N06AX11 | Mirtazapine | 46 | 2.5% |
| N06AX16 | Venlafaxine | 18 | 1.0% |
| N06AX21 | Duloxetine | 8 | 0.4% |
| N06AX26 | Vortioxetine | 1 | 0.1% |

**Table S7:** PIM identified through the application of the Table 2 of Beers 2019 criteria (medications that are potentially inappropriate in most older adults).

| ATC code | PIM independent of diagnosis or condition | PIM frequency  n=916 | PIM % |
| --- | --- | --- | --- |
| A02BC | Proton pump inhibitors | 362 | 39.5% |
| A10BB01 | Glibenclamide | 2 | 0.2% |
| A10BB12 | Glimepiride | 2 | 0.2% |
| B01AC07 | Dipyridamole | 1 | 0.1% |
| C01BD01 | Amiodarone | 6 | 0.7% |
| M01AB05 | Diclofenac | 21 | 2.3% |
| M01AB08 | Etodolac | 20 | 2.2% |
| M01AC01 | Piroxicam | 1 | 0.1% |
| M01AC06 | Meloxicam | 1 | 0.1% |
| M01AE01 | Ibuprofen | 21 | 2.3% |
| M01AE02 | Naproxen | 48 | 5.2% |
| M03BX08 | Cyclobenzaprine | 40 | 4.4% |
| N03AA02 | Phenobarbital | 2 | 0.2% |
| N03AE01 | Clonazepam | 16 | 1.8% |
| N04AA01 | Trihexyphenidyl | 1 | 0.1% |
| N05AD01 | Haloperidol | 1 | 0.1% |
| N05BA01 | Diazepam | 68 | 7.4% |
| N05BA04 | Oxazepam | 16 | 1.8% |
| N05BA05 | Potassium clorazepate | 7 | 0.8% |
| N05BA06 | Lorazepam | 73 | 8.0% |
| N05BA12 | Alprazolam | 121 | 13.2% |
| N05BB01 | Hydroxyzine | 18 | 2.0% |
| N05CD01 | Flurazepam | 2 | 0.2% |
| N05CD04 | Estazolam | 4 | 0.4% |
| N05CD05 | Triazolam | 1 | 0.1% |
| N05CF02 | Zolpidem | 19 | 3.0% |
| N06AA06 | Trimipramine | 1 | 0.1% |
| N06AA09 | Amitriptyline | 24 | 2.6% |
| N06AB05 | Paroxetine | 17 | 1.9% |

**Table S8:** PIM identified through the application of the Table 3 of Beers 2019 criteria (medications that are potentially inappropriate in older adults with certain conditions).

| ATC code | PIM considering disease and syndrome interactions | Diagnose | PIM frequency  n=157 |
| --- | --- | --- | --- |
| A03FA01 | Metoclopramide | Parkinson disease | 3 |
| C08DA01 | Verapamil | Heart failure | 1 |
| C08DB01 | Diltiazem | Heart failure | 1 |
| H02AB13 | Deflazacort | Delirium | 1 |
|  |  | Dementia or cognitive impairment |  |
| M01AB05 | Diclofenac | Heart failure | 3 |
|  |  | History of gastric or duodenal ulcers |  |
|  |  | Chronic kidney disease stage 4 or higher (creatinine clearance <30 mL/min) |  |
| M01AB08 | Etodolac | Heart failure | 3 |
|  |  | History of gastric or duodenal ulcers |  |
|  |  | Chronic kidney disease stage 4 or higher (creatinine clearance <30 mL/min) |  |
| M01AB11 | Acemetacin | Heart failure | 5 |
|  |  | History of gastric or duodenal ulcers |  |
|  |  | Chronic kidney disease stage 4 or higher (creatinine clearance <30 mL/min) |  |
| M01AC01 | Piroxicam | Heart failure | 1 |
|  |  | History of gastric or duodenal ulcers |  |
|  |  | Chronic kidney disease stage 4 or higher (creatinine clearance <30 mL/min) |  |
| M01AE01 | Ibuprofen | Heart failure | 3 |
|  |  | History of gastric or duodenal ulcers |  |
|  |  | Chronic kidney disease stage 4 or higher (creatinine clearance <30 mL/min) |  |
| M01AE02 | Naproxen | Heart failure | 11 |
|  |  | History of gastric or duodenal ulcers |  |
|  |  | Chronic kidney disease stage 4 or higher (creatinine clearance <30 mL/min) |  |
| M01AE03 | Ketoprofen | Heart failure | 2 |
|  |  | History of gastric or duodenal ulcers |  |
|  |  | Chronic kidney disease stage 4 or higher (creatinine clearance <30 mL/min) |  |
| M01AX17 | Nimesulide | Heart failure | 1 |
|  |  | History of gastric or duodenal ulcers |  |
|  |  | Chronic kidney disease stage 4 or higher (creatinine clearance <30 mL/min) |  |
| M03BX08 | Cyclobenzaprine | Lower urinary tract symptoms, benign prostatic hyperplasia | 6 |
| N02AB03 | Fentanyl | History of falls or fractures | 1 |
| N02AJ06 | Codeine and paracetamol | History of falls or fractures | 1 |
| N02AJ13 | Tramadol and paracetamol | History of falls or fractures | 12 |
| N02AX02 | Tramadol | History of falls or fractures | 3 |
| N02AX06 | Tapentadol | History of falls or fractures | 1 |
| N05AD01 | Haloperidol | Dementia or cognitive impairment | 1 |
|  |  | Parkinson disease |  |
| N05AH03 | Olanzapine | Syncope | 4 |
|  |  | Dementia or cognitive impairment |  |
|  |  | Parkinson disease |  |
|  |  | Lower urinary tract symptoms, benign prostatic hyperplasia |  |
| N05AH04 | Quetiapine | Dementia or cognitive impairment | 8 |
| N05AX08 | Risperidone | Dementia or cognitive impairment | 3 |
|  |  | Parkinson disease |  |
| N05BA01 | Diazepam | Delirium | 6 |
|  |  | Dementia or cognitive impairment |  |
|  |  | History of falls or fractures |  |
| N05BA04 | Oxazepam | Delirium | 1 |
|  |  | Dementia or cognitive impairment |  |
|  |  | History of falls or fractures |  |
| N05BA05 | Potassium clorazepate | Delirium | 1 |
|  |  | Dementia or cognitive impairment |  |
|  |  | History of falls or fractures |  |
| N05BA06 | Lorazepam | Delirium | 10 |
|  |  | Dementia or cognitive impairment |  |
|  |  | History of falls or fractures |  |
| N05BA08 | Bromazepam | Delirium | 2 |
|  |  | Dementia or cognitive impairment |  |
|  |  | History of falls or fractures |  |
| N05BA12 | Alprazolam | Delirium | 8 |
|  |  | Dementia or cognitive impairment |  |
|  |  | History of falls or fractures |  |
| N05BA18 | Ethyl loflazepate | Delirium | 2 |
|  |  | Dementia or cognitive impairment |  |
|  |  | History of falls or fractures |  |
| N05BA22 | Cloxazolam | Delirium | 2 |
|  |  | Dementia or cognitive impairment |  |
|  |  | History of falls or fractures |  |
| N05BB01 | Hydroxyzine | Lower urinary tract symptoms, benign prostatic hyperplasia | 2 |
| N05CD01 | Flurazepam | Delirium | 1 |
|  |  | Dementia or cognitive impairment |  |
|  |  | History of falls or fractures |  |
| N05CD09 | Brotizolam | Delirium | 1 |
|  |  | Dementia or cognitive impairment |  |
|  |  | History of falls or fractures |  |
| N05CF02 | Zolpidem | Delirium | 2 |
|  |  | Dementia or cognitive impairment |  |
|  |  | History of falls or fractures |  |
| N06AA09 | Amitriptyline | Syncope | 8 |
|  |  | History of falls or fractures |  |
|  |  | Lower urinary tract symptoms, benign prostatic hyperplasia |  |
| N06AB03 | Fluoxetine | History of falls or fractures | 4 |
| N06AB05 | Paroxetine | History of falls or fractures | 7 |
|  |  | Lower urinary tract symptoms, benign prostatic hyperplasia |  |
| N06AB06 | Sertraline | History of falls or fractures | 8 |
| N06AB10 | Escitalopram | History of falls or fractures | 9 |
| N06AX05 | Trazadone | Syncope | 1 |
|  |  | History of falls or fractures |  |
| N06AX11 | Mirtazapine | Syncope | 5 |
|  |  | History of falls or fractures |  |
| N06AX16 | Venlafaxine | History of falls or fractures | 1 |
| N06AX21 | Duloxetine | History of falls or fractures | 1 |

**Table S9:** PIM identified through the application of the Table 4 of Beers 2019 criteria (medications that should be used with caution).

| ATC code | Drugs to be used with caution in older adults | PIM frequency  n=856 | PIM % |
| --- | --- | --- | --- |
| B01AC06 | Acetylsalicylic acid | 45 | 5.3% |
| B01AE07 | Dabigatran etexilate | 7 | 0.8% |
| B01AF01 | Rivaroxaban | 29 | 3.4% |
| C03BA04 | Chlortalidone | 23 | 2.7% |
| C03BA11 | Indapamide | 52 | 6.1% |
| C03CA01 | Furosemide | 181 | 21.1% |
| C03DA01 | Spironolactone | 34 | 4.0% |
| J01EE01 | Sulfamethoxazole and trimethoprim | 1 | 0.1% |
| N02AX02 | Tramadol | 20 | 2.3% |
| N03AF01 | Carbamazepine | 3 | 0.4% |
| N05AA01 | Chlorpromazine | 1 | 0.1% |
| N05AA06 | Cyamemazine | 2 | 0.2% |
| N05AD01 | Haloperidol | 3 | 0.4% |
| N05AD03 | Melperone | 3 | 0.4% |
| N05AH03 | Olanzapine | 8 | 0.9% |
| N05AH04 | Quetiapine | 31 | 3.6% |
| N05AL03 | Tiapride | 3 | 0.4% |
| N05AL05 | Amisulpride | 14 | 1.6% |
| N05AX08 | Risperidone | 9 | 1.1% |
| N05AX12 | Aripiprazole | 1 | 0.1% |
| N06AA04 | Clomipramine | 9 | 1.1% |
| N06AA06 | Trimipramine | 1 | 0.1% |
| N06AA09 | Amitriptyline | 24 | 2.8% |
| N06AA16 | Dosulepin | 2 | 0.2% |
| N06AA21 | Maprotiline | 2 | 0.2% |
| N06AB03 | Fluoxetine | 36 | 4.2% |
| N06AB04 | Citalopram | 4 | 0.5% |
| N06AB05 | Paroxetine | 17 | 2.0% |
| N06AB06 | Sertraline | 77 | 9.0% |
| N06AB08 | Fluvoxamine | 2 | 0.2% |
| N06AB10 | Escitalopram | 71 | 8.3% |
| N06AX05 | Trazadone | 68 | 7.9% |
| N06AX11 | Mirtazapine | 46 | 5.4% |
| N06AX16 | Venlafaxine | 18 | 2.1% |
| N06AX21 | Duloxetine | 8 | 0.9% |
| N06AX26 | Vortioxetine | 1 | 0.1% |

**Table S10:** Potentially drug-drug interactions identified through the application of the Table 5 of Beers 2019 criteria (potentially clinically important drug-drug interactions that should be avoided in older adults).

| Object drug and class | Interacting drug and class | Interactions frequency  n=206 | PIM % |
| --- | --- | --- | --- |
| Opioids | Benzodiazepines | 28 | 13.6% |
|  | Gabapentin, pregabalin | 13 | 6.3% |
| Anticholinergic | Anticholinergic | 14 | 6.8% |
| Antidepressants (TCAs, SSRIs, and SNRIs)  Antipsychotics  Antiepileptics  Benzodiazepines and  nonbenzodiazepine, benzodiazepine  receptor agonist hypnotics  (ie, “Z-drugs”)  Opioids | Any combination of three  or more of these  CNS-active drugs | 109 | 52.9% |
| Corticosteroids, oral or parenteral | NSAIDs | 38 | 18.5% |
| Peripheral α-1 blockers | Loop diuretics | 2 | 1.0% |
| Warfarin | Amiodarone | 1 | 0.5% |
|  | NSAIDs | 1 | 0.5% |

**Table S11:** Medications that should be avoided or have their dosage reduced with varying levels of kidney function in older adults identified through the application of the Table 6 of Beers 2019 criteria.

| ATC code | Medications that should be avoided or have their dosage reduced with varying levels of kidney function in older adults | PIM frequency  n=17 | PIM % |
| --- | --- | --- | --- |
| B01AF01 | Rivaroxaban | 5 | 29.4% |
| B01AF02 | Apixaban | 1 | 5.9% |
| B01AF03 | Edoxaban | 7 | 41.2% |
| C03DA01 | Spironolactone | 1 | 5.9% |
| N03AX14 | Levetiracetam | 3 | 17.7% |

**Table S12:** PIM identified through the application of the Table 7 of Beers 2019 criteria (drugs with strong anticholinergic properties).

| ATC code | Drugs with strong anticholinergic properties | PIM frequency  n=154 | PIM % |
| --- | --- | --- | --- |
| G04BD02 | Flavoxate | 13 | 8.4% |
| G04BD04 | Oxybutynin | 5 | 3.3% |
| G04BD08 | Solifenacin | 5 | 3.3% |
| G04BD09 | Trospium | 12 | 7.8% |
| M03BX08 | Cyclobenzaprine | 40 | 26.0% |
| N04AA01 | Trihexyphenidyl | 1 | 0.7% |
| N05AA01 | Chlorpromazine | 1 | 0.7% |
| N05AH03 | Olanzapine | 8 | 5.2% |
| N05BB01 | Hydroxyzine | 18 | 11.7% |
| N06AA04 | Clomipramine | 9 | 5.8% |
| N06AA06 | Trimipramine | 1 | 0.7% |
| N06AA09 | Amitriptyline | 24 | 15.6% |
| N06AB05 | Paroxtine | 17 | 11.0% |

**Table S13:** Total number of PIM dispensed, according to STOPP v2 criteria.

| ATC code | PIM STOPP v2 | PIM frequency  n=980 | PIM % |
| --- | --- | --- | --- |
| A02BC02 | Pantoprazole | 4 | 0.4% |
| A02BC05 | Esomeprazole | 3 | 0.3% |
| A03FA01 | Metoclopramide | 3 | 0.3% |
| A10BA02 | Metformin | 4 | 0.4% |
| A10BB01 | Glibenclamide | 2 | 0.2% |
| A10BB12 | Glimepiride | 2 | 0.2% |
| B01AC04 | Clopidogrel | 3 | 0.3% |
| B01AC05 | Ticlopidine | 8 | 0.8% |
| B01AC18 | Triflusal | 1 | 0.1% |
| B03AB09 | Ferric proteinsuccinylate | 1 | 0.1% |
| B03AD03 | Ferrous sulfate and folic acid | 1 | 0.1% |
| C01DA14 | Isosorbide mononitrate | 1 | 0.1% |
| C03BA04 | Chlortalidone | 7 | 0.7% |
| C03BA11 | Indapamide | 3 | 0.3% |
| C03CA01 | Furosemide | 28 | 2.9% |
| C03DA01 | Spironolactone | 19 | 1.9% |
| C07AA05 | Propranolol | 2 | 0.2% |
| C07AA07 | Sotalol | 1 | 0.1% |
| C07AB07 | Bisoprolol | 3 | 0.3% |
| C08CA01 | Amlodipine | 2 | 0.2% |
| C08CA13 | Lercanidipine | 1 | 0.1% |
| C09AA03 | Lisinopril | 1 | 0.1% |
| C09AA04 | Perindopril | 3 | 0.3% |
| C09AA05 | Ramipril | 1 | 0.1% |
| C09CA04 | Irbesartan | 1 | 0.1% |
| C09CA08 | Olmesartan medoxomil | 1 | 0.1% |
| G04BD02 | Flavoxate | 2 | 0.2% |
| G04BD04 | Oxybutynin | 2 | 0.2% |
| G04BD08 | Solifenacin | 1 | 0.1% |
| G04BD09 | Trospium | 4 | 0.4% |
| H02AB13 | Deflazacort | 2 | 0.2% |
| H02BX01 | Methylprednisolone, combinations | 1 | 0.1% |
| M01AB05 | Diclofenac | 25 | 2.6% |
| M01AB08 | Etodolac | 23 | 2.4% |
| M01AB11 | Acemetacin | 34 | 3.5% |
| M01AB16 | Aceclofenac | 3 | 0.3% |
| M01AB55 | Diclofenac, combinations | 3 | 0.3% |
| M01AC01 | Piroxicam | 2 | 0.2% |
| M01AC06 | Meloxicam | 1 | 0.1% |
| M01AE01 | Ibuprofen | 32 | 3.3% |
| M01AE02 | Naproxen | 54 | 5.5% |
| M01AE03 | Ketoprofen | 4 | 0.4% |
| M01AH01 | Celecoxib | 6 | 0.6% |
| M01AH05 | Etoricoxib | 23 | 2.4% |
| M01AX17 | Nimesulide | 4 | 0.4% |
| M03BX08 | Cyclobenzaprine | 11 | 1.1% |
| M05BA04 | Alendronic acid | 7 | 0.7% |
| M05BA06 | Ibandronic acid | 2 | 0.2% |
| M05BB03 | Alendronic acid and colecalciferol | 6 | 0.6% |
| N02AA55 | Oxycodone and naloxone | 1 | 0.1% |
| N02AB03 | Fentanyl | 5 | 0.5% |
| N02AJ13 | Tramadol and paracetamol | 94 | 9.6% |
| N02AX02 | Tramadol | 15 | 1.5% |
| N02AX06 | Tapentadol | 17 | 1.7% |
| N05AA01 | Chlorpromazine | 1 | 0.1% |
| N05AD01 | Haloperidol | 3 | 0.3% |
| N05AD03 | Melperone | 3 | 0.3% |
| N05AH03 | Olanzapine | 8 | 0.8% |
| N05AH04 | Quetiapine | 31 | 3.2% |
| N05AL03 | Tiapride | 3 | 0.3% |
| N05AL05 | Amisulpride | 14 | 1.4% |
| N05AX08 | Risperidone | 9 | 0.9% |
| N05AX12 | Aripiprazole | 1 | 0.1% |
| N05BA01 | Diazepam | 68 | 6.9% |
| N05BA04 | Oxazepam | 16 | 1.6% |
| N05BA05 | Potassium clorazepate | 7 | 0.7% |
| N05BA06 | Lorazepam | 73 | 7.5% |
| N05BA08 | Bromazepam | 32 | 3.3% |
| N05BA09 | Clobazam | 3 | 0.3% |
| N05BA12 | Alprazolam | 121 | 12.4% |
| N05BA18 | Ethyl loflazepate | 23 | 2.4% |
| N05BA22 | Cloxazolam | 9 | 0.9% |
| N05BB01 | Hydroxyzine | 18 | 1.8% |
| N05CD01 | Flurazepam | 2 | 0.2% |
| N05CD04 | Estazolam | 4 | 0.4% |
| N05CD05 | Triazolam | 1 | 0.1% |
| N05CD08 | Midazolam | 4 | 0.4% |
| N05CD09 | Brotizolam | 5 | 0.5% |
| N05CD11 | Loprazolam | 1 | 0.1% |
| N05CF02 | Zolpidem | 19 | 1.9% |
| N06AA04 | Clomipramine | 1 | 0.1% |
| N06AA09 | Amitriptyline | 5 | 0.5% |
| N06AB05 | Paroxetine | 4 | 0.4% |
| N07CA02 | Cinnarizine | 1 | 0.1% |
